# Mechanical Tillage Diversely Affects Glomalin Content, Water Stable Aggregates and AM Fungal Community in the Soil Profiles of Two Differently Managed Olive Orchards

**DOI:** 10.3390/biom9100639

**Published:** 2019-10-22

**Authors:** Luca Lombardo, Assunta Maria Palese, Filomena Grasso, Donald H. Duffy, Caterina Briccoli Bati, Cristos Xiloyannis

**Affiliations:** 1 Center Agriculture Food Environment (C3A), University of Trento, 38122 Trento, Italy; 2 Research and Innovation Centre, Fondazione Edmund Mach, 38010 San Michele all’Adige, Italy; 3 Ages s.r.l.s. Spin-off Accademico, Università degli Studi della Basilicata, 85100 Potenza, Italy; palesedina@gmail.com; 4 Dipartimento di Agraria, Università degli Studi di Napoli “Federico II”, 80055 Portici, Italy; filo.grasso2@gmail.com; 5 Department of Computer Science and Automation Control, University of Salamanca, 37007 Salamanca, Spain; dhduffy@usal.es; 6 Ketensis, New York, NY 10036, USA; 7 CREA Research Centre for Olive, Citrus and Tree Fruit, 87036 Rende, Italy; cbbati@yahoo.com; 8 Department of European and Mediterranean Cultures, Architecture, Environment and Cultural Heritage (DiCEM), Università degli Studi della Basilicata, 75100 Matera, Italy; cristos.xiloyannis@unibas.it

**Keywords:** glomalin, water stable aggregates, conventional system, sustainable system

## Abstract

This work was designed to investigate the effect of mechanical tillage on glomalin content, arbuscular mycorrhizal fungi (AMF) abundance and diversity, and the concentration of water stable aggregates (WSA), in two adjacent olive groves located in Basilicata (Italy) that were managed over the course of 11 years in accordance with different horticultural models (conventional and sustainable). Soil sampling was performed at four depths between the trees within a row and between rows. In the end, WSA was found to be a highly sensitive indicator (especially in the “macro” fraction) of the effect of management on soil structure, showing the highest statistically significant values within the sustainable system. In the same regard, the diversity of the AM fungal community was negatively affected by conventional practices; on the other hand, a higher concentration of glomalin in the first 20 cm layer of the conventional system is here reported for the first time, as a likely result of disruption of the mycelium provoked by the mechanical tillage.

## 1. Introduction

Glomalin is a high molecular weight insoluble glycoprotein stored in great quantity in the cell wall of the hyphae of arbuscular mycorrhizal fungi (AMF) [1], whose production shows a certain degree of seasonality [2] and whose turnover rate in soils has been estimated to range from 6 to 42 years [3,4,5]. Once released outside, it is able to improve soil fertility by slowing the degradation of organic matter and the associated nutrient loss through the stabilization, via hydrophobic interactions, of aggregates that physically protect the particulate matter from the activity of enzymes [6,7,8,9,10]. In this regard, glomalin concentration in agricultural soils has been reported to positively correlate with soil content of organic carbon (SOM) [11] and water-stable aggregates (WSA) [12,13,14], whereas aggregate stability represents an indicator of organic matter content, biological activity, and nutrient cycling in soil. As sustainable soil management in agro-ecosystems has been widely demonstrated to improve the water regime, limit the leaching of nutrients, ameliorate root development, increase the porosity of the soil, and stimulate microorganisms’ activity by increasing the resilience of communities and establishing a better telluric air/water rate [15,16], the aim of the present study was to evaluate the effects of two different agronomic management systems -sustainable (SS) versus conventional (CS)- on glomalin content, AMF diversity, WSA, and some of the major factors affecting chemical, physical, and biological soil fertility in an Italian olive grove.

## 2. Materials and Methods

### 2.1. Experimental Olive Grove

**Figure 1:**
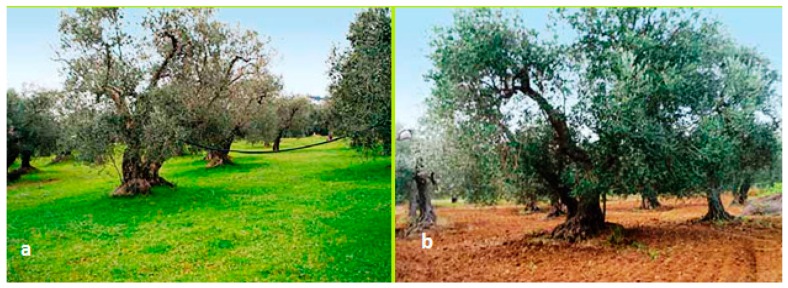
Sustainable -SS- (**a**) and Conventional -CS- (**b**) management systems in the two adjacent olive orchards.

The research was conducted in an experimental olive grove located at Ferrandina, Matera province, Italy (40°29′ N, 16°28′ E), which consists of mature vase-shaped trees, cultivar Maiatica, with an irregular planting pattern of about 8 × 8 m. The soil of the experimental grove was a sandy loam, classified as a Haplic Calcisol [17], with a mean bulk density of 1.5 t m^−3^. The orchard (about 6600 m^2^), managed in accordance with to eco-compatible farming techniques, included a contiguous non-irrigated parcel managed according to local traditional agricultural techniques (Figure 1). The sustainable farming model (SS) provided no till farming, spontaneous cover crops, and light annual pruning, with resulting material left in the field and guided fertilization. The conventional system (CS) provided plants growing under rain-fed conditions, shallow tillage, mineral fertilization with ternary fertilizers carried out once a year, and biennial heavy pruning with removal of pruned material. Soil tillage was performed by means of a tractor-mounted disc harrow. 

Since 2000, the sustainable olive grove was equipped with a drip irrigation system spreading municipal wastewater treated by a pilot unit according to simplified schemes [18] adjacent to the city depurator. The reclaimed wastewater was generally distributed from May to October by drip irrigation (six self-compensating drippers per plant delivering 8 L h^−1^). 

The chemical characteristics of the treated wastewater are reported in Table 1.

Soil samples were taken at depths of 0–20, 20–40, 40–60, and 60–80 cm using a core sampler of 5 cm in diameter, in springtime, a week after tillage. Six sampling points per each management system and position were chosen according to the scheme: 

Sustainable system:

- Under the dripper, in the wet area during the irrigation season, (2 m from the trunk in the row) (SS_r_);
- Inter-rows (4 m from the trunk between the rows) where crop residues and pruning material were left on the ground as mulch (SS_i_).
- Conventional system:
- 2 m from the trunk of olive trees in the row (CS_r_);
- Inter-rows (4 m from the trunk between the rows) (CS_i_).

### 2.2. Soil Analysis

Soil analyses were performed in triplicate in accordance with the official methods (DM 11/05/1992 and DM No. 79, No. 185 13/09/1999) aimed at assessing fertility levels and possible limiting factors such as deficiencies in elements or toxic amounts thereof, deriving from the use of treated municipal wastewater.

The amount of total organic carbon (TOC) was evaluated by the Walkley and Black method [19] using Potassium dichromate 1 N as oxidizing agent in presence of sulfuric acid. The organic matter content (OM) was then calculated by multiplying the amount of carbon for the empirical factor set equal to 1.72 [20]. Total nitrogen and phyto-available phosphorus were determined through the Kjeldahl [21] and Olsen [22] methods, respectively. 

Soil moisture was determined gravimetrically, while a pH-meter was used for potentiometric pH measurements of a soil-water suspension at a ratio 1:2. The cation-exchange capacity (CEC) was calculated adding to 2.5 g of soil sample 50 mL of a solution of barium chloride (BaCl_2_) and triethanolamine buffered at pH 8.1. The soil-barium complex thereby obtained was stirred for 1 h and then filtered with Whatman^TM^ No. 42 filters. The filtrate was then diluted 1:10 and analyses of exchangeable bases (Ca, Mg, K, and Na) were performed by ICP-OES Spectrometer (iCAP 6000 Series, Thermo Scientific, Waltham, MA, USA). 

### 2.3. Glomalin and Water Stable Aggregates

Glomalin soil content can be evaluated through two predominant fractions: easily extractable glomalin (EEG; the newly formed one) and total glomalin (TG; the stable and persistent form, which is more difficult to extract). Total and easily extractable glomalin were extracted, according to Wright and Upadhyaya [23]. Glomalin is insoluble in water and the extraction of this protein requires harsh conditions indicative of the stability of said molecule. Total glomalin (TG) was extracted from 1 g of soil in 8 mL of 50 mM sodium citrate (pH 8) in repeated 1 h-cycles in autoclave at 121 °C until the supernatant was almost colorless. Easily extractable glomalin (EEG) was extracted at 121 °C for 30 min in 20 mM citrate (pH 7). 

As the extraction method has been proved to be not totally specific for glomalin, this protein mixture (comprising also humic acids) is often referred to as glomalin-related soil protein (GRSP) [8]. The easily extractable glomalin should represent the newly formed and most active fraction of the protein, but some objections have been raised. The extracts were centrifuged at 10,000× *g* to remove soil particles, and then quantified by spectrophotometric readings (NanoDrop 2000, Thermo Scientific) at 595 nm with the Bio-Rad Protein Assay based on the Bradford method [24], using bovine serum albumin (BSA) as internal standard. Each sample was extracted twice and subjected to two readings per extraction. 

Water-stable aggregates (WSA) were measured by wet sieving, according to Kemper and Rosenau [25] modified, on stacked sieves with mesh of 710, 400, 250, 100, and 53 µm in diameter, using 20 g of soil immersed in distilled water for 30 min. Oven-dried aggregates were grouped in macroaggregates (≥ 250 µm) and microaggregates (< 250 µm) in accordance with Oades and Waters [26]. 

All data were subjected to multifactorial ANOVA and Tukey’s HSD test by Statgraphics Plus 5.1 vers. (Managistics Inc., New York, NY, USA) software.

### 2.4. Spore Isolation and Molecular Characterization of Autochthonous AMF

AMF spores were isolated from 100 g air-dried soil samples by wet sieving. The soil retained in each sieve (400, 200, 100, and 50 μm in diameter) was diluted in a 40% sucrose solution and centrifuged at 5000× *g* for 15 min. After centrifugation, spores, and spore clusters contained in the filtered supernatant were then transferred into Petri dishes and counted in three replications under stereomicroscope at 100× magnification. Only visually intact spores were counted and sorted into categories based on size, shape and color. Single spores were separated and washed with distilled water. Spores were then crushed with a sterilized mini-pestle for 30 seconds and incubated for 5 minutes at room temperature. The DNA was then resuspended in 50 μL of TE buffer (10 mM Tri-HCL, pH 8, 1 mM EDTA). Partial 18S rDNA fragments of AM fungi were amplified by nested PCR. NS1 (5′-GTA GTC ATA TGC TTG TCT C-3′) and NS4 (5′-CTT CCG TCA ATT CCT TTA AG-3′) universal primers [27] were used in the first amplification followed by a nested amplification with AM fungal specific primers AML1 and AML2 (5′-CCA AAC ACT TTG GTT TCC-3′) [28]. PCR products of the expected size (~800 bp) were checked and purified from agarose gel with GeneJET Gel Extraction Kit (Thermo Fisher Scientific), cloned using the pGEM-T vector system (Promega) and transformed into *Escherichia coli* (Xl1 blue). Clones containing the transformed vector were then sequenced at a minimum of 12 to a maximum of 20 clones per sample. DNA strands were sequenced on automatic sequencer ABI Prism 310^®^ (Applied Biosystems, Foster City, CA, USA). Query sequences were identified through BLASTn. MUSCLE v.3.8.31 [29] was used for multiple alignment of DNA sequences and a phylogenetic tree was built using the iTOL v4 online database [30]. After alignment, the obtained sequences were clustered in operative taxonomic units (OTUs) with a 97% similarity level threshold 

AMF species diversity was evaluated through the Shannon’s index *H* [31]:

(1) $$H=-\overset{S}{\underset{i=1}{\sum }}p_{i}lnp_{i}$$

Shannon’s index is a measure of diversity which takes into account the absolute as well as the relative (evenness) abundance of species present, with *S* being the total number of species (richness) at this site and *p_i_* (proportional number of the *i-th* species) = Ni/N, with Ni = abundance of the *i-th* species and N = abundance of the total number of species. As species abundancy and eveness increase, so too does diversity, whilst a value of 0 would represent a community with just one species. 

Eventually, a measure of species evenness was developed using the Shannon equitability index *E_H_* [31]:

(2) $$E_{H}=H/H_{max}=H/lnS$$

This parameter defines how close in numbers each species in an environment is. Equitability assumes a value between 0 and 1, with 1 being complete evenness (equal number of every species) and values next to 0 revealing the presence of a dominant species.

## 3. Results 

### 3.1. Soil Analysis 

Soil characteristics are reported in Table 2. The pH values were all in the alkaline range (pH 7.91–8.55) and were uniformly distributed throughout the soil profile in the two treatments.

Regardless of the treatment, organic matter, P, and N concentrations were highly significantly superior (*p* < 0.01) in the first 20 cm layer. According to the management system, moisture and CEC values were statistically significantly higher in the conventional system treatment at confidence level of 99% and 95% respectively; however, no difference was found in the first 20 cm. Within the same parcel, no significant difference was found in the CS treatment in relation to the sampling point, while in the SS treatment, electrical conductivity (EC) and cation exchange capacity (CEC) were statistically higher (*p* < 0.05) in the SSr position (between the trees in the row, under the dripper), presenting even the highest concentrations of the base cations Mg^2+^, and Na^+^. 

### 3.2. Glomalin and Water Stable Aggregates 

**Figure 2:**
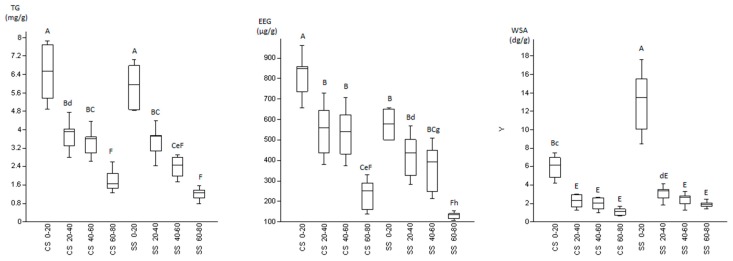
Box plot and Tukey HSD intervals for the pairwise comparisons of total glomalin (TG)/depth, easy extractable glomalin (EEG)/depth and water stable aggregates (WSA)/depth, according to the management system. CS: conventional system; SS: sustainable system. Different capital letters and lowercase indicate a statistical difference at *p* < 0.01 and *p* < 0.05 level respectively.

As expected, the highest statistically significant (*p* < 0.01) values of WSA, EEG, and TG were recorded in the surface layer (0–20 cm) and the lowest in the deepest layer (>60 cm; Table 3 and Figure 2). Regarding the management system, higher, but not statistically significant, values of TG and statistically significantly higher values of EEG were found in the 20 cm layer in the orchard that was managed conventionally. On the other hand, the amount of WSA was significantly (*p* < 0.01) higher in the olive orchard that was managed according to a sustainable model (Figure 2). In both treatments WSA were represented for the most part by macro-aggregates (> 250 µm), accounting for 69–78% of the total (Table 3). 

Lastly, by comparing the positions (between the trees in the line and between the lines) within the same parcel, no significant differences were found for WSA, TG, and EEG. 

### 3.3. AMF Spore Abundance, Distribution and Identification

**Figure 3:**
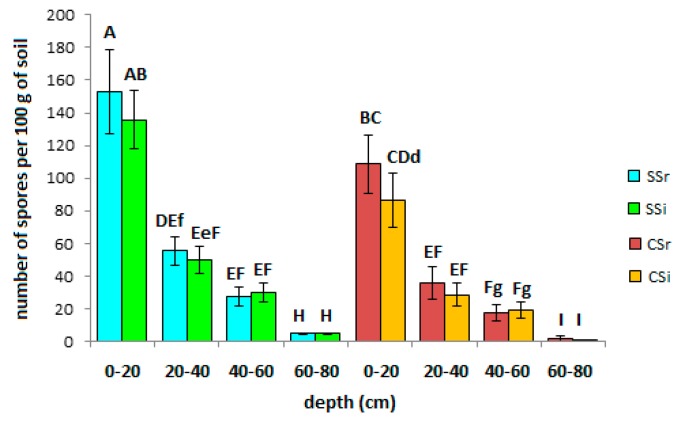
Spore abundance expressed as number of spores per 100 g of soil with standard deviation and Tukey HSD intervals. CSr: conventional system in the row; CSi: conventional system inter-rows; SSr: sustainable system in the row; SSr: sustainable system inter-rows. Different capital letters and lowercase indicate a statistical difference at *p* < 0.01 and *p* < 0.05 level, respectively.

The statistically significantly higher number of spores was found in the first 20 cm of the sustainable system both in the inter-rows (on average 153 spores per gram of soil) and between the rows (on average 135 spores per gram of soil; Figure 3), confirming olive as a highly mycotrophic species [32,33]. Spore abundance drastically decreased in the lower layers, up to only a few (1–4) spores over 60 cm deep.

**Figure 4:**
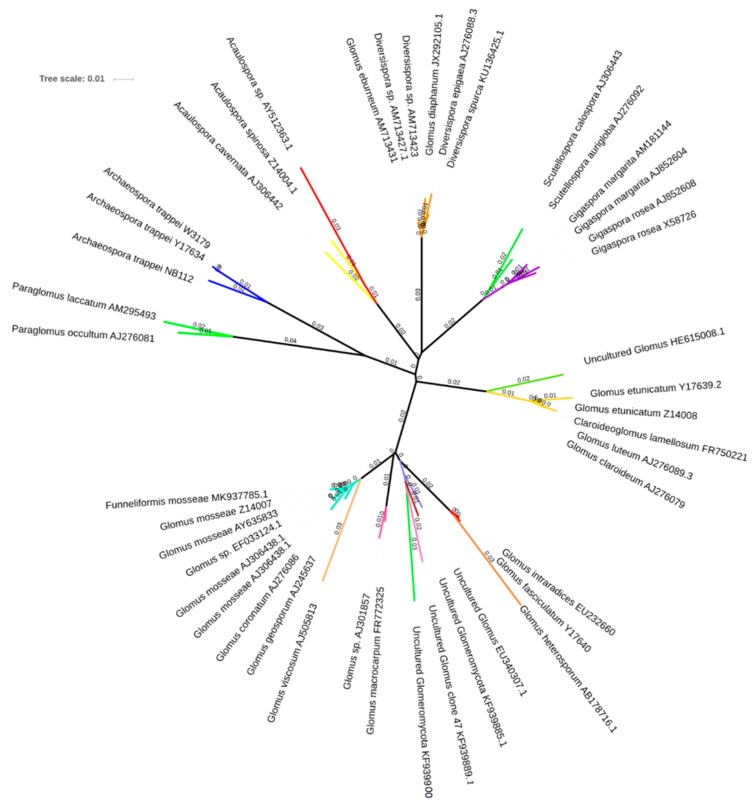
Neighbor-joining consensus phylogram for partial 18S rDNA sequence of arbuscular mycorrhizal fungal spores. Different colors indicate different OTUs at a 97% similarity threshold. Numbers next to the nodes indicate the branch length.

After alignment of the sequences obtained through the molecular analyses, the spores were attributed to a total of 18 OTUs (Figure 4), belonging for the most part—55%—to Glomerales (Glomeraceae and Claroideoglomeraceae). The remaining OTUs were composed of Diversisporales (Diversisporaceae, Acaulosporaceae, Scutellosporaceae, and Gigasporaceae) and Paraglomerales (Paraglomeraceae). 

The number of spores from each OTU was used to evaluate AM fungal community diversity in the two management systems. The Shannon index, in the end, was higher in the sustainable system, particularly in the row (*H* = 2.53 vs. 2.26 between the rows), than the conventional one (*H* -CSr- = 1.85; *H* -CSir-= 1.77). On the other hand, Shannon’s equitability (*E_H_*) assumed practically identical values in the two parcels, being equal to 0.91 in the SSr, SSir, and CSir as well as being equal to 0.89 in the CSr.

**Figure 5:**
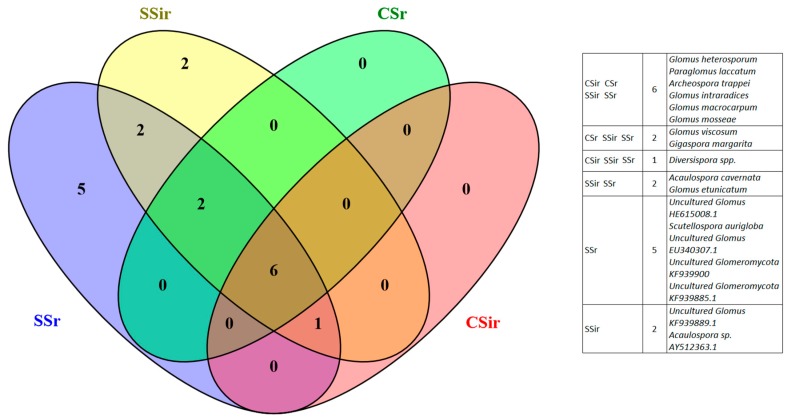
Venn diagram reporting the distribution of the identified operative taxonomic units (OTUs), each represented by the most abundant morphotype.

The higher diversity in the sustainable system was further highlighted by the number of species that occurred in only one (singletons) and two (doubletons) sampling points. Specifically, 5 OTUs were exclusively present in SSi and 2 in the SSir, while 2 OTUs were exclusively present in SSi and SSir, as shown in the Venn diagram (Figure 5) reporting the most abundant morphotypes from each OTU. 

### 3.4. Principal Component Analysis

**Figure 6:**
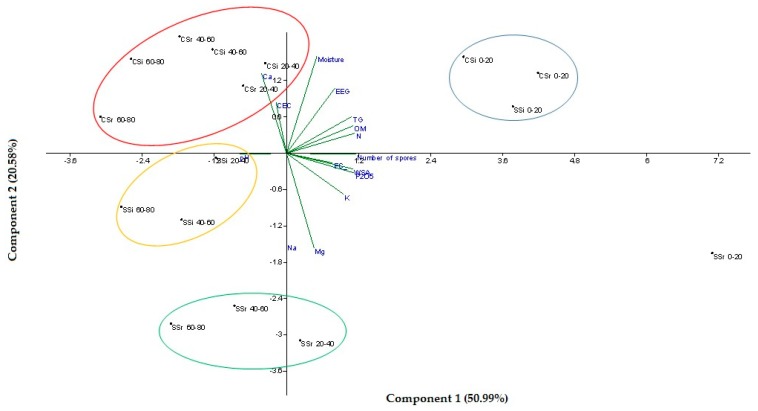
Principal Component Analysis of the measured variables in relation to soil management, distance from the tree, and soil depth.

The overall effect of soil management, distance from the tree and soil depth on all the measured variables was evaluated through a Principal Component Analysis (PCA, Figure 6). A clear distinction between the first 20 cm layer and the deeper layers was highlighted, Regarding the position in the upper layer, SSr did not cluster with CSr, CSi, and SSi because of the highest values of WSA, Mg, Na, P, and P_2_O_5_, as well as the number of spores found in this sampling position. In the lower layers, the employment of treated wastewater for irrigation allowed us to discriminate between the SSr and the SSi treatment, while the different management system was responsible for the separation of the SS and Cs clusters.

## 4. Discussions 

### 4.1. Soil Analysis

Regardless of the treatment used, organic matter, P, and N concentrations were significantly superior (*p* < 0.01) in the first 20 cm layer. According to the management system, moisture and CEC values were statistically significantly higher in the conventional system at confidence levels of 99 and 95% respectively; however, no difference was found in the first 20 cm. This was probably due to the effects of tillage that was carried out just one week before soil sampling, during which a weak rain event occurred. Tillage has been demonstrated to improve the rate of water infiltration and intake, thus influencing soil moisture in the shallow layers of soil [34,35]. 

Organic matter content in the first 20 cm layer was quite high in both treatments, and, despite not being statistically significant, it was 10% higher on average in the cover cropped plots. In particular, in the inter-rows, as a likely result of mulching with crop residues and pruning material, the increase of OM was of around 3.5 g kg^−1^.

These findings, together with the higher values of P and N, were related to the fertigation with treated wastewater (Table 1), which proved to be an efficient alternative to chemical fertilizers in a sustainable management of agro-environments, despite a net increase in soil concentrations of Mg and Na, that did not seem to negatively affect soil fertility. As a proof of this, according to previous studies carried out in the same orchards, wastewater-irrigated olive trees had a constant and higher yield and improved fruit characteristics with no dangerous faecal contamination [36], while only a moderate amount of total heavy metals was recorded in the soil, well below the quantity of metals which can be distributed yearly by sewage sludge according to the pertinent Italian law [37].

### 4.2. Glomalin and Water Stable Aggregates 

Soil glomalin concentration fell within the range described in other works (typically, from 2 to 15 mg/g to over 60 mg/g; [3,38]). Regarding the management system, higher values of glomalin were found in the first layer of the conventionally managed orchard (Table 3 and Figure 2). This was probably due to the disruption of the mycelium provoked by the periodical mechanical tillage causing the release of the protein contained in the cell walls. This fact is supported by the statistically significantly higher values of EEG, the newly formed fraction, which is likely to be leaked from cell walls after mechanical tillage, which was performed only a few days before sampling. To our knowledge, this is the first time this evidence has been reported, as previous studies found lower glomalin concentrations in the switching from uncultivated to cultivated soils and from no-tilled to conventional-tilled soils [39,40,41,42,43,44]. 

The large amount of glomalin found in the conventional system can also explain the high value of organic carbon found in this parcel. In fact, glomalin, being composed of about 30–40% of carbon which is stored for the most part in the N-linked oligosaccharides, can incisively contribute to the pool of this element [23], representing up to the 5% of the total C [3,40] and even up to 35% of the organic fraction in the soil (Nichols et al., 2004). It is also a good source of nitrogen [3,6,38], reportedly representing up to 5% of the total soil N in the 0–10 cm soil layer [45].

On the contrary, the amount of WSA was significantly higher in the olive orchard managed according to a sustainable model (*p* < 0.01, Figure 2), because of the greater stability of the soil structure conferred by cover crops and no-till farming. In particular, primary soil particles free of bonds and silt-sized aggregates (<20 μm) are bound together in micro-aggregates (20–250 μm) by persistent binding agents (e.g., humified organic matter and polyvalent metal complexes), oxides and aluminosilicates. These stable micro-aggregates, in turn, are bound together in macro-aggregates (> 250 μm) by temporary bonds (e.g., fungal hyphae and roots) and by transient bonds (e.g., polysaccharides of microbial and plant origin). Following this hierarchical order of aggregates and their binding agents, the stability of the macro-aggregates appears to be lower and more dependent on the agronomic management than that of the micro-aggregates [46]. The higher the number of WSA is, the lower the risk is of soil erosion due to the beating action of the rain and surface runoff, breaking aggregates into smaller particles of soil forming the surface crust [47]. In this sense, aggregate stability is often used as a measurement of soil structure [48]. The different soil aggregate fractions provide spatially heterogeneous conditions for microbes, including differences in SOM composition, oxygen concentration, and water potential, and these conditions can change the soil microbial community compositions [49]. Accordingly, a no-tillage practice has been reported to lead to increased aggregate stability, while soil aggregation generally affects the stability of SOM in upper soil horizons [49,50,51,52].

### 4.3. AMF Spore Abundance, Distribution, and Identification

Spore abundance throughout the soil profiles can be linearly related with TG and EEG, although, in the first 20 cm layer, the effect of soil tillage was here suggested to alter this correlation, even because of the slow turnover of this protein, that can remain in the soil for several years [3,4,5]. At the same time, mechanical tillage is likely responsible of the statistically significant difference in spore density between the two systems found in the first 20 cm layer. This because the disruption of the mycelium provoked by the periodical mechanical tillage may have a detrimental effect on the total biomass of AM fungi. On the other hand, the presence of an irrigation system in the SS may have had a positive effect on AMF biomass as highest spore number and percent colonization of AM fungi were previously recorded in irrigated sites [53,54]. In the same regard, molecular analyses allowed us to conclude that the diversity of the AM fungal community in olive soils is negatively affected by conventional practices, as was previously reported for other crop cultures [55,56]. This was confirmed by the Shannon-Weaver index, which showed higher results for the sustainable system. Regarding species evenness, the slight differences found between the two management systems suggest that although conventional practices provoked a considerable decrease in the number of species, some species tended to adapt to adverse conditions and maintained a state of equilibrium in the whole AMF community structure. Moreover, spore identification showed that Glomerales represented the predominant group of AMF, which have been reported to commonly dominate in many different environments [32,57,58,59,60].

Eventually, despite nitrogen and phosphorus addition in soil having previously been described to reduce AMF spore population diversity and richness [61,62,63,64], no clear effects have been recorded here; the concentrations distributed by the treated wastewater (and to a lesser extent via ternary fertilizers) were probably not sufficient to induce an obvious negative effect, as confirmed by previous work conducted in these experimental groves reporting significantly more culturable fungi and bacteria, greater metabolic diversity indices of microbial communities, and greater soil enzyme activities in the SS than in the CS [65].

## 5. Conclusions

Cover crops have been confirmed to play an important role in sustainable agriculture due to their functions in improving soil structure, nutrient cycling, and overall microbial activity. Furthermore, the use of treated municipal wastewater has proved to be an efficient alternative to chemical fertilizers. Water stable aggregates were a highly sensitive indicator of the effect of soil management on soil structuration, showing statistically significantly higher values in the sustainable farming model. Accordingly, conventional practices negatively affected the abundance and diversity of AMF, which was likely mainly because of chemical fertilization and mechanical tillage. On the other hand, a higher concentration of glomalin in the first 20 cm layer of the conventional system is reported here for the first time, as a likely result of the disruption of the mycelium provoked by the mechanical tillage, which turned out to be likely responsible for the high organic carbon content recorded in this soil management system.

## Figures and Tables

**Table 1:** Chemical parameters of treated municipal wastewater.

Parameter	Unit of Measure	Value
pH		7.6
Conductivity	µS cm^−1^	884
Na	mg L^−1^	121.3
Mg	mg L^−1^	13.8
Ca	mg L^−1^	67.8
N (NO_3_^-^)	mg L^−1^	18.3
N (NH_4_^+^)	mg L^−1^	0.0
B	mg L^−1^	1.0
K	mg L^−1^	17.0
P	mg L^−1^	1.0
COD	mg L^−1^	180

COD: chemical oxygen demand.

**Table 2:** Soil chemical and physical characteristics of the experimental olive orchards (values ±standard deviation).

Treatment	Depth (cm)	pH	EC (µS/cm)	N (%)	P (ppm)	P_2_O_5_ (ppm)	CEC (meq/100g)	Base Cations (meq/100g)				Moisture (%)	OM (%)
								Ca	Mg	K	Na		
**CSr**	0-20	8.19 ± 1.10	140.00 ± 17.98	0.13 ± 0.03	18.71 ± 2.08	42.85 ± 8.44	14.15 ± 1.62	12.24 ± 2.41	0.55 ± 0.08	1.09 ± 0.14	0.27 ± 0.04	13.23 ± 3.39	2.61 ± 0.61
	20-40	8.33 ± 1.08	117.67 ± 15.73	0.09 ± 0.01	6.77 ± 0.82	15.51 ± 3.19	12.39 ± 1.38	11.45 ± 2.11	0.38 ± 0.06	0.35 ± 0.06	0.21 ± 0.03	12.94 ± 2.76	1.75 ± 0.47
	40-60	8.43 ± 1.07	116.33 ± 15.58	0.08 ± 0.01	4.93 ± 0.62	11.29 ± 2.37	16.35 ± 1.76	15.18 ± 2.47	0.44 ± 0.08	0.41 ± 0.07	0.32 ± 0.04	13.09 ± 3.06	1.51 ± 0.48
	60-80	8.47 ± 1.06	121.33 ± 16.08	0.05 ± 0.01	3.80 ± 0.46	8.71 ± 1.79	16.10 ± 1.69	14.88 ± 2.43	0.48 ± 0.08	0.29 ± 0.06	0.35 ± 0.04	12.24 ± 2.89	1.47 ± 0.46
**CSi**	0-20	8.55 ± 1.05	109.33 ± 14.87	0.12 ± 0.02	19.07 ± 2.10	43.67 ± 8.57	12.36 ± 1.43	11.00 ± 2.04	0.36 ± 0.06	0.78 ± 0.10	0.22 ± 0.03	13.52 ± 3.31	2.55 ± 0.63
	20-40	8.29 ± 1.09	120.67 ± 16.03	0.09 ± 0.01	7.63 ± 0.91	17.48 ± 3.56	13.09 ± 1.45	12.15 ± 2.18	0.32 ± 0.06	0.37 ± 0.06	0.25 ± 0.03	12.48 ± 2.82	1.80 ± 0.48
	40-60	8.21 ± 1.10	126.33 ± 16.61	0.06 ± 0.01	4.43 ± 0.54	10.13 ± 2.09	14.60 ± 1.56	13.58 ± 2.33	0.39 ± 0.06	0.39 ± 0.06	0.24 ± 0.03	11.98 ± 2.77	1.63 ± 0.45
	60-80	8.32 ± 1.08	112.33 ± 15.19	0.07 ± 0.01	3.12 ± 0.42	7.14 ± 1.56	15.76 ± 1.69	14.68 ± 2.43	0.46 ± 0.07	0.35 ± 0.06	0.28 ± 0.04	12.77 ± 2.90	1.39 ± 0.45
**SSr**	0-20	7.91 ± 1.14	173.00 ± 21.31	0.16 ± 0.04	24.68 ± 2.72	56.52 ± 11.10	13.15 ± 1.57	10.11 ± 2.02	1.58 ± 0.21	0.99 ± 0.15	0.47 ± 0.06	13.03 ± 3.42	2.71 ± 0.61
	20-40	8.34 ± 1.08	135.00 ± 17.46	0.07 ± 0.03	10.57 ± 1.17	24.21 ± 4.76	12.60 ± 1.37	9.88 ± 1.95	1.28 ± 0.18	0.93 ± 0.14	0.51 ± 0.06	8.42 ± 2.31	1.53 ± 0.40
	40-60	8.46 ± 1.06	118.50 ± 15.80	0.07 ± 0.03	8.94 ± 1.01	20.47 ± 4.06	13.38 ± 1.45	10.79 ± 2.02	1.21 ± 0.17	0.85 ± 0.14	0.52 ± 0.06	9.66 ± 2.45	1.59 ± 0.43
	60-80	8.47 ± 1.06	115.67 ± 15.51	0.06 ± 0.03	8.49 ± 0.95	19.45 ± 3.84	13.40 ± 1.44	11.01 ± 2.05	1.15 ± 0.17	0.69 ± 0.12	0.55 ± 0.06	9.27 ± 2.41	1.41 ± 0.40
**SSi**	0-20	8.18 ± 1.10	110.67 ± 15.04	0.12 ± 0.02	12.98 ± 1.49	29.72 ± 5.95	12.97 ± 1.49	11.02 ± 2.08	0.48 ± 0.07	1.23 ± 0.15	0.24 ± 0.04	12.78 ± 2.98	2.93 ± 0.60
	20-40	8.40 ± 1.07	89.67 ± 12.92	0.06 ± 0.01	8.61 ± 0.96	19.72 ± 3.89	11.81 ± 1.28	10.65 ± 2.02	0.37 ± 0.06	0.55 ± 0.08	0.24 ± 0.03	10.42 ± 2.50	1.50 ± 0.42
	40-60	8.29 ± 1.09	85.33 ± 12.50	0.06 ± 0.01	8.32 ± 0.93	19.04 ± 3.76	10.46 ± 1.14	9.43 ± 1.91	0.35 ± 0.06	0.45 ± 0.07	0.23 ± 0.03	8.30 ± 2.15	1.28 ± 0.36
	60-80	8.39 ± 1.07	78.00 ± 11.75	0.06 ± 0.01	6.06 ± 0.70	13.88 ± 2.79	11.20 ± 1.22	10.12 ± 1.97	0.40 ± 0.07	0.43 ± 0.07	0.25 ± 0.03	10.38 ± 2.33	1.40 ± 0.39

EC: electrical conductivity; CEC: cation exchange capacity; OM: organic matter; CSr: conventional system in the row; CSi: conventional system inter-rows; SSr: sustainable system in the row; SSr: sustainable system inter-rows.

**Table 3:** Average water stable aggregates (WSA), total (TG), and easily extractable (EEG) glomalin concentrations measured in the two fields per position and depth.

Treatment	Depth (cm)	TG (mg/g)	EEG (μg/g)	WSA (dg/g)	Macro-Aggregates (%)
**CSr**	0–20	6.53 ± 1.16 ^a^	757.75 ± 101.25 ^a,b^	6.16 ± 1.33 ^c^	76.33
	20–40	3.36 ± 0.57 ^d,e^	438.26 ± 58.14 ^defg^	2.31 ± 0.66 ^d,e^	72.84
	40–60	3.11 ± 0.49 ^d,e,f^	432.14 ± 57.13 ^d,e,f,g^	2.03 ± 0.64 ^d,e^	71.23
	60–80	1.46 ± 0.19 ^e,f^	159.23 ± 21.36 ^h,i^	1.40 ± 0.29 ^d,e^	70.97
**CSi**	0–20	6.37 ± 1.48 ^a^	849.19 ± 112.52 ^a^	5.57 ± 1.40 ^c,d^	77.38
	20–40	4.03 ± 0.72 ^b,c,d^	643.50 ± 85.27 ^b,c^	2.13 ± 0.85 ^d,e^	71.66
	40–60	3.68 ± 0.68 ^cd^	623.63 ± 82.63 ^b,c,d^	1.82 ± 0.80 ^d,e^	73.08
	60–80	2.11 ± 0.50 ^d,e,f^	290.54 ± 38.43 ^f,g,h,i^	0.72 ± 0.06 ^e^	69.13
**SSr**	0–20	5.96 ± 1.09 ^a,b^	579.53 ± 78.40 ^b,c,d^	15.55 ± 2.05 ^a^	78.16
	20–40	3.75 ± 0.63 ^b,c,d^	325.96 ± 44.50 ^e,f,g,h^	3.49 ± 0.61 ^c,d,e^	75.51
	40–60	2.46 ± 0.46 ^d,e,f^	248.60 ± 33.54 ^g,h,i^	2.83 ± 0.50 ^d,e^	72.42
	60–80	1.37 ± 0.21 ^e,f^	123.39 ± 16.79 ^i^	1.88 ± 0.18 ^d,e^	70.88
**SSi**	0–20	5.81 ± 0.98 ^a,b,c^	575.37 ± 75.73 ^c,d^	10.07 ± 1.60 ^b^	72.01
	20–40	3.08 ± 0.64 ^d,e,f^	501.58 ± 65.78 ^c,d,e^	2.60 ± 0.79 ^d,e^	75.56
	40–60	2.26 ± 0.53 ^d,e,f^	450.06 ± 58.52 ^c,d,e,f^	1.95 ± 0.70 ^d,e^	70.54
	60–80	1.03 ± 0.24 ^f^	134.66 ± 17.86 ^h,i^	1.93 ± 0.53 ^d,e^	73.26

CSr: conventional system in the row; CSi: conventional system inter-rows; SSr: sustainable system in the row; SSr: sustainable system inter-rows. Different lowercase letters indicate a statistical difference at *p* < 0.05.
